# Members of the XB3 Family from Diverse Plant Species Induce Programmed Cell Death in *Nicotiana benthamiana*

**DOI:** 10.1371/journal.pone.0063868

**Published:** 2013-05-22

**Authors:** Xiaoen Huang, Xueying Liu, Xiuhua Chen, Anita Snyder, Wen-Yuan Song

**Affiliations:** 1 Department of Plant Pathology, University of Florida, Gainesville, Florida, United States of America; 2 Plant Molecular and Cellular Biology Program, University of Florida, Gainesville, Florida, United States of America; 3 Institute of Genetics and Developmental Biology, Chinese Academy of Sciences, Beijing, China; Virginia Tech, United States of America

## Abstract

Programmed cell death has been associated with plant immunity and senescence. The receptor kinase XA21 confers resistance to bacterial blight disease of rice (*Oryza sativa*) caused by *Xanthomonas oryzae* pv. *oryzae* (*Xoo*). Here we show that the XA21 binding protein 3 (XB3) is capable of inducing cell death when overexpressed in *Nicotiana benthamiana*. XB3 is a RING finger-containing E3 ubiquitin ligase that has been positively implicated in XA21-mediated resistance. Mutation abolishing the XB3 E3 activity also eliminates its ability to induce cell death. Phylogenetic analysis of XB3-related sequences suggests a family of proteins (XB3 family) with members from diverse plant species. We further demonstrate that members of the XB3 family from rice, Arabidopsis and citrus all trigger a similar cell death response in *Nicotiana benthamiana*, suggesting an evolutionarily conserved role for these proteins in regulating programmed cell death in the plant kingdom.

## Introduction

The self-defense of plants against various and ever-evolving pathogens has resulted in the evolution of multiple layers of the plant immune system. The first layer of this complex system often consists of membrane-spanning receptors capable of recognizing highly conserved molecules from pathogens, known as pathogen-associated molecular patterns (PAMPs), and a number of downstream defense regulators [1]. Activation of this signaling chain results in PAMP-triggered immunity (PTI). Examples of well-characterized plant pattern-recognition receptors (PRRs) include the Arabidopsis flagellin sensitive 2 (FLS2) and elongation factor receptor (EFR), which recognize 22- and 18-amino acid peptides (flg22 and elf18) derived from bacterial flagellin and elongation factor Tu (EF-Tu), respectively [2], [3].

To overcome plant PTI, some pathogens have developed effector proteins with diverse molecular features, a subset of which can directly suppress PTI signaling [4], [5]. For pathogenic bacteria, effectors are delivered into plant cells by the type III secretion systems (T3SSs) where they play key roles in promoting disease. As a counter response, some plant cultivars have reestablished resistance through recognition of the pathogen effectors by resistance (R) proteins [1]. Effector-triggered immunity (ETI) is often associated with strong immune responses, including the hypersensitive response (HR), which is characterized by localized, rapid cell death at infection sites [6]. In addition to ETI, transient overexpression of a number of *R* genes or defense regulators can also induce pathogen-independent cell death reminiscent of HR in *Nicotiana benthamiana* (*N. benthamiana*) [7]–[11].

The rice gene *Xa21* was originally identified as an *R* gene, conferring resistance to *Xanthomonas oryzae pv. oryzae* (*Xoo*), the causal agent of bacterial blight disease of rice [12]. *Xa21* encodes a receptor kinase protein that recognizes a sulfated peptide (axY^S^22) embedded in the N-terminal region of the bacterial polypeptide Ax21 [13]. Because of wide conservation of Ax21 in the genus *Xanthomonas*, XA21-mediated defense is thought to be a part of PTI.

XA21 interacts through its intracellular kinase domain (XA21K) with a number of rice proteins [14]–[20]. One of them, XA21 binding protein 3 (XB3), consists of eight imperfect ankyrin repeats at the N-terminal half, a RING (Really Interesting New Gene) finger (RF) motif, and a C-terminal tail (XB3-C) that potentially forms a coil-coil structure [14]. *In vitro* protein pull-down assays reveal that the ankyrin repeats are sufficient for binding to XA21K, whereas biochemical analyses indicate that the RF domain of XB3 acts as an active E3 ubiquitin ligase. Down-regulation of the *Xb3* gene in rice by RNA interference significantly destabilizes the XA21 protein and compromises *Xa21*-mediated disease resistance. Therefore, we proposed that XB3 functions as a positive regulator of XA21 immunity [14]. However, the question of whether XB3 could activate defense responses remains to be addressed. It is also unclear whether the E3 ubiquitin ligase activity of XB3 is required for its function.Here we show that XB3 is a member of a highly conserved E3 ubiquitin ligase family. Overexpression of XB3 or other members of this E3 family from rice, Arabidopsis, and citrus induces a cell death response in *N. benthamiana*.

## Materials and Methods

### Phylogenetic Analysis

Protein sequences were retrieved from NCBI by using the XB3 sequence as a query and aligned using CLUSTAL W [21]. The neighbor-joining phylogenetic tree was constructed using the MEGA4 program [22]–[23]. Bootstrap values were obtained by 1000 bootstrap replicates.

### Bacteria and Plant Growth Conditions

*Agrobacterium tumefaciens* strain EHA105 was grown at 29°C in Luria-Bertani medium with appropriate antibiotics. *N. benthamiana* was grown at 24–26°C with a 16 hour photoperiod under florescent light.

### Constructs

The XB3 overexpression construct pC1300S-XB3 was made by cloning the PCR amplified *Xb3* gene (primer pair Xb3 nb-1/Xb3 nb-2) into the *BamH*I site of the binary vector pCAMBIA1300S. All the primers used in this study are listed in Table S1. A similar method was used to construct pC1300S-XB3^C323A^ (primer pair Xb3nb-1/Xb3nb-2).

To fuse a 3xFLAG epitope tag in frame to the C-terminus of XB3, the *Xb3* gene was PCR amplified using the primer pair Xb3NEW-1/Xb3NEW-3 and cloned into the pCR8GW-3xFLAG vector for sequencing. The resultant *Xb3* was then subcloned into the *Xba*I site of the pCAMBIA1300S binary vector. A similar strategy was used to construct pCAMBIA1300S- XB3ΔC-3xFLAG (primer pair XB3New-1/XB3New-7), pCAMBIA1300S- XB3Ank-3xFLAG (primer pair XB3New-1/XB3New-5), pCAMBIA1300S- XB3RFC-3xFLAG (primer pair XB3New-6/XB3CT-3), pCAMBIA1300S- XB3RF-3xFLAG (primer pair XB3New-6/XB3New-7), pCAMBIA1300S- XB3C-3xFLAG (primer pair XB3New-13/XB3CT-3), pCAMBIA1300S-XBOS31-3xFLAG (primer pair Xbos31nb-5/Xbos31nb-6), pC1300S-XBAT31-3xFLAG (primer pair Xbat31nb-1/Xbat31nb-1), pC1300S-XBAT32-3xFLAG (primer pair Xbat32nb-1/Xbat32nb-1), pCAMBIA1300S-XBCT31-3xFLAG (primer pair Xbct31nb-1/Xbct31nb-2), and pCAMBIA1300S-XBCT32-3xFLAG (primer pair 041054 m-3/041054 m-4). All constructs were confirmed by sequencing.

### *Agrobacterium*-mediated Transient Assay in *N. benthamiana*

*Agrobacterium*-mediated transformation of *N. benthamiana* was performed according to Wroblewski et al. [24] with modification. Overnight bacterial cultures carrying the constructs of interest were harvested by centrifugation at 4,000 *g* for ten minutes. Harvested cells were resuspended into buffer (10 mM MES, pH 5.6, 10 mM MgCl_2_ and 150 µM acetosyringone) and adjusted to an OD_600_ of 0.5. Following incubation at room temperature for three hours, infiltration of 4-week-old *N. benthamiana* leaves was performed using a 1-ml needleless syringe. Cell death phenotypes were scored at 2 and 3 dpi (days post infiltration), unless indicated otherwise. Tissue samples were collected at 40 hpi (hours post infiltration) for protein extraction.

### Protein Blot Analysis

Protein blot analysis was performed as described previously [14] using a modified protein extraction buffer: [50 m M Tris-HCl, pH 7.4, 150 m M NaCl, 10% glycerol, 0.5% TritonX-100, 2 mM EDTA, 2% polyvinylpolypyrrolidone (PVPP), 2 mM DTT, 1 mM phenylmethylsulfonyl fluoride].

### Electrolyte Leakage Measurement

To quantify cell death, electrolyte leakage was performed according to [25]. Three leaf discs (∼10 mm in diameter) were collected from the *Agrobacterium*-infiltrated area and immersed in 10 mL of non-ionic, double-distilled water. After incubation at room temperature for two hours with shaking at 160 rpm, conductivity of the solution was measured using a COND 6^+^ conductivity meter (EUTECH Instruments). Error bars represent three replicates at each time point.

## Results

### Sequence Analysis of the XB3 Family Members

**Figure 1:**
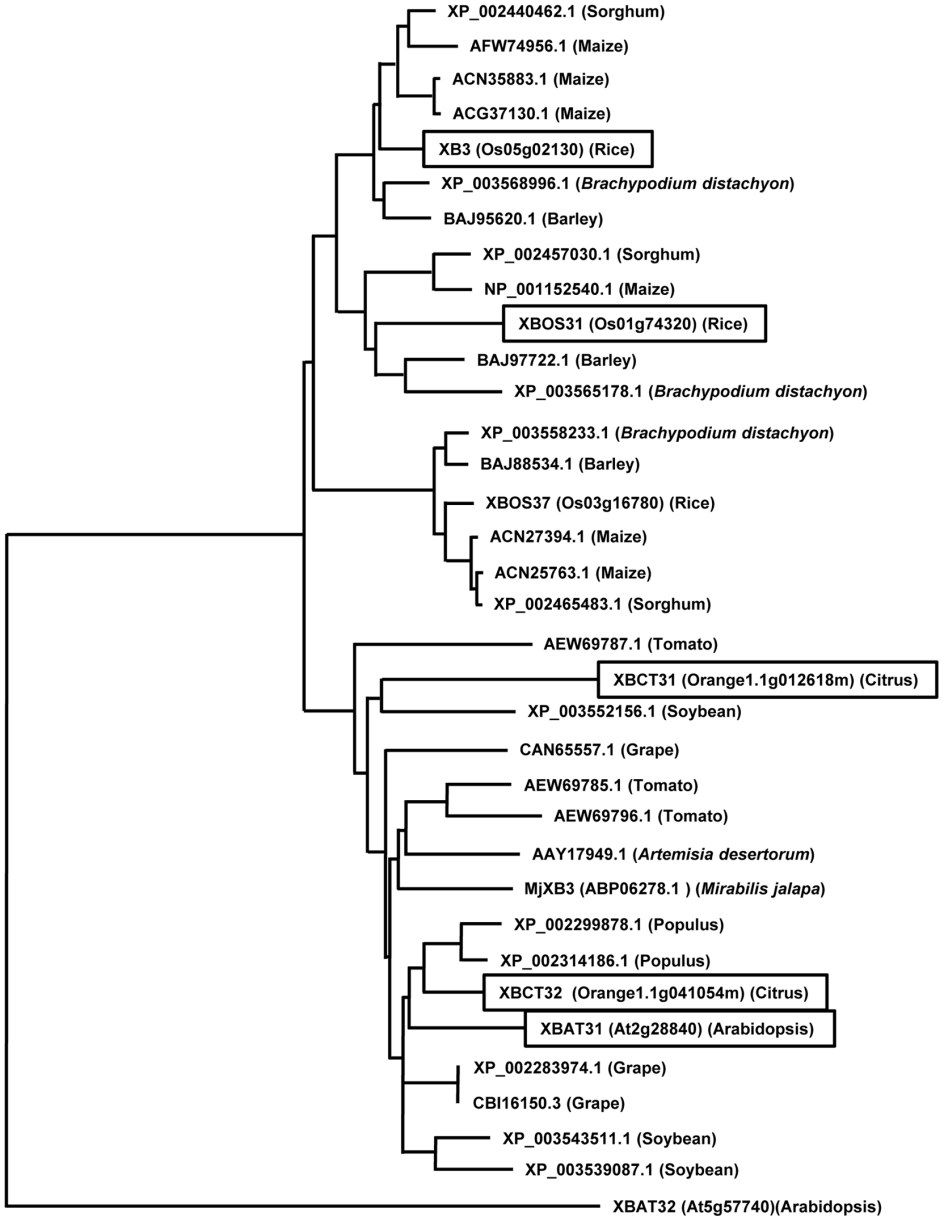
Phylogenetic analysis of the XB3 family. The phylogenetic tree was generated by the neighbor-joining method. Accession numbers for the sequences are indicated. The functionally characterized XB3 family members in this study are boxed.

To identify proteins functionally related to XB3, we searched the databases with the BLASTP program. A total of 58 proteins were identified from diverse plant species ranging from rice and Arabidopsis (annual) to woody citrus (perennial). All of the identified sequences share similar ankyrin-RING structures. Phylogenetic analysis revealed that 34 out of the 58 identified proteins, including XB3, form a large group with two major subclades that differentiate sequences from dicotyledonous and monocotyledonous plants (Figure 1). This group of proteins was apparently more related to XB3, and therefore named the XB3 family. The copy number of the XB3 family members varies among plant species. For instance, rice contains three members, XB3, XBOS31 and XBOS37, whereas Arabidopsis carries only one, XBAT31. In the newly sequenced citrus genome [26], [27], we identified two XB3 family members, XBCT31 and XBCT32. The XB3 family is phylogenetically distinct from XBAT32, an ankyrin-RING protein that has been implicated in lateral root development [28].

### The Rice XB3 Protein can Elicit Cell Death in *N. benthamiana*

**Figure 2:**
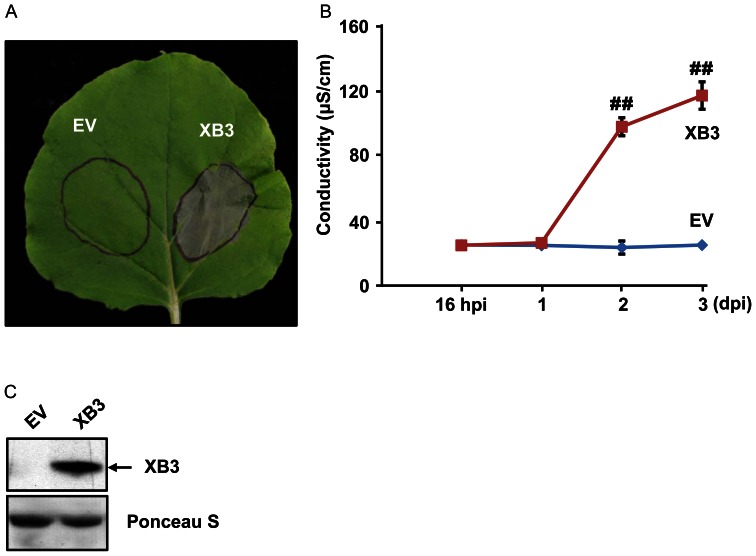
Cell death triggered by XB3. (**A**) Indicated proteins were expressed in *N. benthamiana* leaves by infiltration of *Agrobacterium* (agroinfiltration) carrying the corresponding constructs. Infiltrated areas are circled. Photograph was taken 3 days post infiltration. EV: empty vector. (**B**) Quantification of XB3-triggered cell death. Electrolyte leakage was measured in the infiltrated leaves at the indicated time points after agroinfiltration (hpi: hours post infiltration, dpi: days post infiltration). Each data point represents the mean+SD from 3 infiltrated leaves. Data sets with pound signs indicate statistically significant differences from the control (EV at 16 hpi) as calculated by Student's t test (##: P<0.01). (**C**) Protein blot analysis showing the level of XB3 in the infiltrated leaves. Total protein extracts were prepared 40 hpi and immunoblotted with anti-XB3 M antibodies (Top). The same blot stained with Ponceau S to show sample loading (Bottom).

To determine whether XB3 is capable of activating a cell death response, we overexpressed this protein in *N. benthamiana*. This heterologous plant species has a well-established transient overexpression system mediated by *Agrobacterium* transformation (agroinfiltration) [29]–[30] and is ideal for cell death assays. Furthermore, we assumed that the XB3 function in plant immunity might be evolutionarily conserved based on the phylogenetic analysis described above. An *Xb3* construct, driven by the cauliflower mosaic virus (CaMV) 35S promoter, was delivered into 4-week-old *N. benthamiana* leaves by agroinfiltration. The infiltrated areas started developing visible grey patches at day 2 (48 hours) and completely collapsed three days after agroinfiltration (Figure 2A). This necrotic phenotype was reminiscent of HR caused by avirulent pathogen infections. As a control, infiltration of *Agrobacterium* containing the empty vector (pCAMBIA1300S) failed to induce tissue death. To confirm XB3 accumulation, we carried out protein blot analyses using previously developed anti-XB3 M antibodies that can specifically detect XB3 in rice [14]. Leaf samples were harvested 40 hours after infiltration, at which time the necrotic phenotype was not apparent. XB3 was detected in the leaf discs infiltrated with the *Xb3* construct, but not in those with the empty vector (Figure 2B). These results indicated that the overexpressed rice XB3 triggers cell death in *N. benthamiana*.

Cell death is often associated with electrolyte leakage caused by membrane damage [8]. Ion leakage assays were performed to quantify XB3-induced cell death. As shown in Figure 2C, significant amounts of ion leakage were detected 48 and 72 hours after infiltration with the *Xb3* harboring *Agrobacterium*. In the infiltrated leaf discs, the development of visible tissue collapse kinetically correlated with the time course of ion leakage. Ion leakage was not induced by agroinfiltration of the empty vector. These data confirm the cell death activity of XB3.

### The RF Domain of XB3 is Required for the Cell Death Response

**Figure 3:**
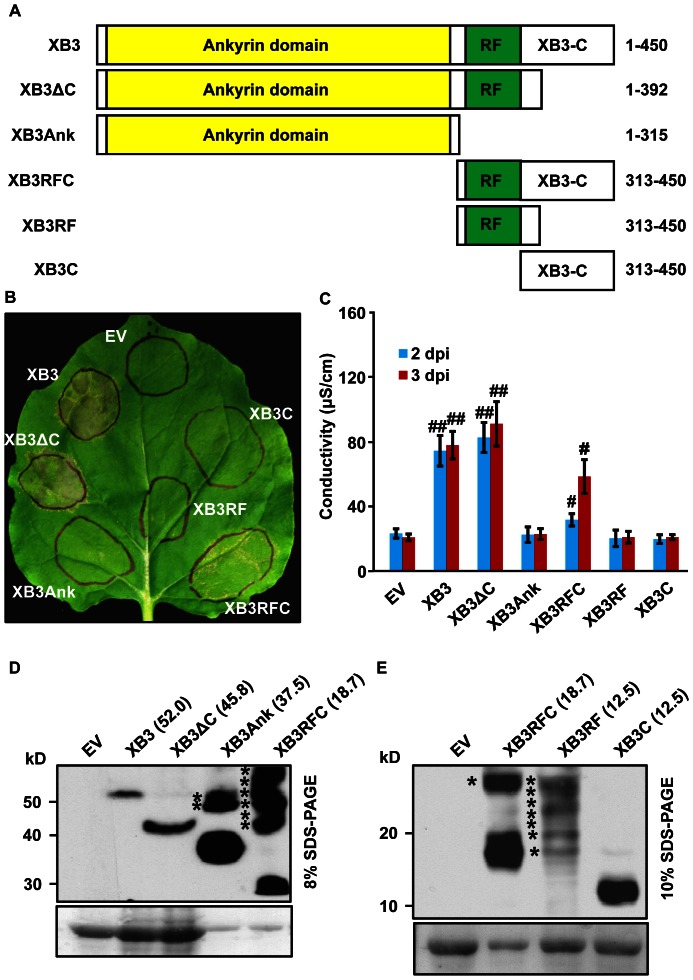
The RING finger (RF) motif of XB3 is required for its cell death activity. (A) Schematic diagram showing structure of XB3 and its truncation mutants used for agroinfiltration. Domains were as described by [14]. Numbers on the left indicate amino acid positions of each product in the full-length XB3. A 3xFLAG epitope tag was individually fused to the C-terminus of each protein. (B) Phenotypes induced by the expression of XB3 or its truncation mutants in *N. benthamiana* leaves. (**C**) Quantification of the cell death induced by the indicated proteins. Agroinfiltration and electrolyte leakage assays were performed as described in the Figure 2 legend. Data sets with pound signs indicate statistically significant differences from the control (EV) as calculated by Student's t test (#: P<0.05; ##: P<0.01). (D, E) Protein blot analyses showing the levels of XB3 and its truncation mutants in the infiltrated leaves. Total protein extracts were prepared 40 hours after infiltration and immunoblotted with anti-FLAG M2 antibody (Top). The same blot was stained with Ponceau S to show sample loading (Bottom). The theoretical molecular weights (kDa) of each protein are shown in parentheses. Percentages of the SDS-PAGE gels used for resolving the proteins are indicated. Asterisks denote high molecular weight products derived from the indicated mutants.

As mentioned above, XB3 mainly consists of three domains: eight ankyrin repeats, a RF motif, and a XB3-C [14]. We have previously shown that the ankyrin repeats of XB3 acts as a protein-protein interaction domain capable of binding to the kinase region of XA21 [14]. To define the minimal sequence for cell death activity, different domains of XB3 or combinations of domains (Figure 3A) were transiently expressed in *N. benthamiana* leaves. Both tissue collapse and ion leakage were used as criteria for the cell death response. For protein detection, a 3xFLAG epitope tag was fused to the C-terminus of XB3 and its derivatives. Expression of the tagged XB3 (full-length) induced a similar cell death phenotype as that of wild-type XB3, indicating that XB3-3xFLAG is functional (Figure 3B and 3C). The XB3ΔC mutant, lacking the C-terminal coil-coil domain, triggered a similar or even slightly higher level of cell death as compared with XB3. Another mutant retaining cell death activity was XB3RFC, which encompasses the RF motif and the XB3-C domain. Unlike XB3 or XB3ΔC, XB3RFC elicited only a partial response. No single domains (the ankyrin domain, the RF motif, or XB3-C alone) induced measurable cell death.

To confirm the expression of the XB3 truncations in the infiltrated leaves, protein blot analyses were carried out using anti-FLAG M2 antibody. This antibody detected a single product with the expected size of XB3-3xFLAG from the leaves infiltrated with the corresponding construct, but not from the empty vector control (Figure 3D). Since XB3-3xFLAG and its FLAGged truncation variants encompass a wide range of molecular weights, ranging from 52.0 to 12.5 kDa, proteins were resolved using an 8% or 10% SDS-PAGE gel. The XB3RFC-3xFLAG sample was included in both gels for pattern comparisons. Notably, most of the XB3 truncations, including XB3ΔC-3xFLAG, XB3Ank-3xFLAG, XB3RFC-3xFLAG, and XB3C-3xFLAG, accumulated to much higher levels than XB3-3xFLAG did (Figure 3D and 3E). Almost no protein with the expected size of XB3RF-3xFLAG was detected (Figure 3E). In addition to the XB3 variants, judged by the expected molecular weight of each protein, products with higher molecular weights were observed in the samples of XB3Ank-3xFLAG, XB3RFC-3xFLAG, and XB3RF-3xFLAG. These products likely originate from the XB3 variants, possibly representing post-translational modifications of these altered proteins.

### The E3 Deficient Mutant XB3^C323A^ is Unable to Induce Cell Death

**Figure 4:**
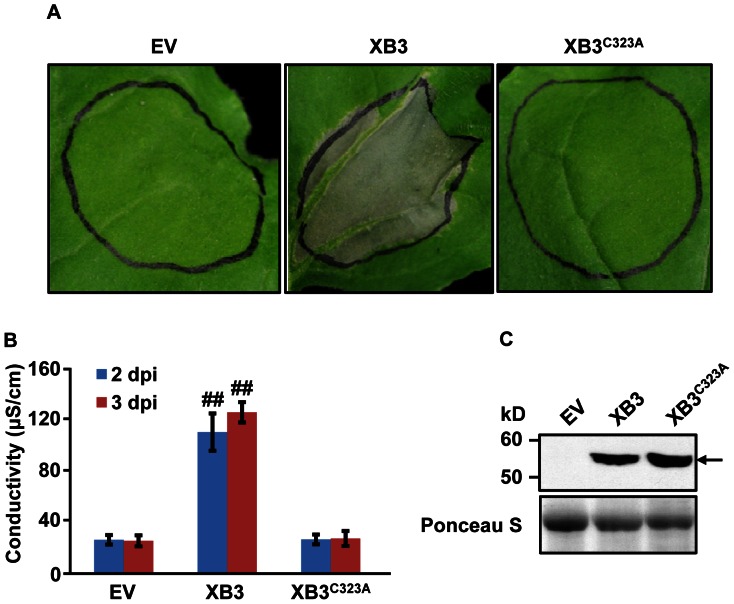
The E3 ubiquitin ligase deficient mutant XB3^C323A^ is unable to induce cell death. Phenotypes (**A**) and electrolyte leakage (**B**) induced by the expression of XB3 or XB3^C323A^ in *N. benthamiana* leaves. Data sets with pound signs indicate statistically significant differences from the control (EV) as calculated by Student's t test (##: P<0.01). (C) Accumulation of the XB3 or XB3^C323A^ protein in the infiltrated leaf discs. All assays were performed as described in the Figure 2 legend.

Mutation of the conserved Cys323 residue abolishes XB3 autoubiquitination activity [14]. We asked whether this mutation also eliminates XB3 cell death activity in *N. benthamiana*. Although the XB3^C323A^ protein was expressed to a level comparable to or even higher than wild-type XB3 (Figure 4B), the mutant failed to trigger any visible cell death (Figure 4A). This observation was further supported by ion leakage assays (Figure 4C). Thus, the RF motif is essential for XB3 cell death activity, and the XB3 E3 ubiquitin ligase activity is likely required for this function.

### Members of the XB3 Family from Rice, Arabidopsis, and Citrus can All Induce Cell Death

**Figure 5:**
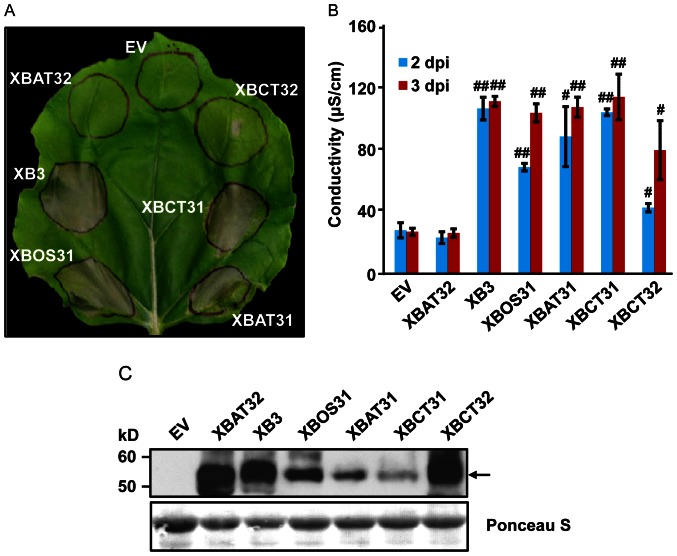
Cell death triggered by members of the XB3 family. A 3xFLAG epitope tag was individually fused to the C-terminus of the indicated protein family members from rice (XB3, XBOS31), Arabidopsis (XBAT31) and citrus (XBCT31 and XBCT32) and the fusion proteins were expressed in *N. benthamiana* leaves. The Arabidopsis protein XBAT32 with the same tag and the empty vector (EV) was used as negative controls. Leaf phenotypes (**A**), electrolyte leakage (**B**) and protein accumulation (C) were determined as described in the Figure 3 legend. Data sets with pound signs indicate statistically significant differences from the control (EV) as calculated by Student's t test (#, P<0.05; ##, P<0.01).

Given the closely-related domain structure and sequences (Figure 1), we wanted to determine whether other members of the XB3 family share cell death activity. The *XBOS31* gene was cloned from rice previously [14]. We isolated, using RT-PCR, the genes encoding XBAT31, XBCT31, and XBCT32 from Arabidopsis and citrus, respectively. Because XB3-3xFLAG functions as wild-type XB3 in cell death induction, we assumed that the addition of the same tag at the C-termini would not interfere with the potential cell death function of other XB3 members. As expected, when expressed in leaves by the CaMV 35 promoter, these proteins all elicited tissue collapse to various degrees (Figure 5A). *Xb3* and *Xbct31* appeared to be stronger cell death inducers, whereas *Xbct32* seemed to have a weaker activity. In addition to the empty vector, the Arabidopsis *Xbat32* gene, encoding an active E3 ubiquitin ligase [28], did not induce any visible tissue collapse. Data from ion leakage assays were consistent with the above observations (Figure 5B). Anti-FLAG M2 antibody detected the expected XB3 family members in the infiltrated leaf discs (Figure 5C). Notably, the abundance of each protein in the leaves did not correlate with the intensity of tissue death symptoms. For example, XBCT31 accumulated to a significantly lower level as compared with XBCT32. However, a much more severe tissue death was induced by XBCT31. Despite these differences, our results indicated that the XB3 family represents a conserved cell death-inducing function.

## Discussion

Reduction-of-expression studies have suggested a positive role for XB3 in XA21-mediated immunity [14]. The data presented here show that XB3 alone is capable of eliciting a cell death response when overexpressed in *N. benthamiana*. This activity is specific because the Arabidopsis protein XBAT32 with a similar ankyrin-RING finger structure but low sequence identity (30%), is unable to trigger cell death. Accordingly, the only known role for XBAT32 is the regulation of lateral root development [28]. The specificity of XB3 cell death activity is further supported by the inability of a series of XB3 mutants to trigger cell death.

HR-like cell death in *Nicotiana* species is a hallmark for defense activation by various R proteins and their regulators. For example, coexpression of the tomato *Pto* with its cognate *Pseudomonas syringae AvrPto* genes induces a leaf necrosis [29], [31]. Transient coexpression of the bacterial protease AvrPphB with the Arabidipsis R protein RPS5 and its binding kinase PBS1 initiates cell death [32]. In some scenarios, HR-like responses can be activated by R proteins or their down-stream signaling proteins in the absence of pathogen effectors. Well-demonstrated examples include the Arabidopsis and barley R proteins RPS2, RPS4, and MLA10, and a number of mitogen-activated kinase kinase kinases from tomato or *N. benthamiana* [7]–[11], [33]–[34]. These phenomena are thought to be the result of constitutive activation of downstream defense responses due to a stochiometric overabundance of these proteins. Therefore, cell death triggered by overexpression of XB3 might be indicative of an activation of defense mechanisms, which is consistent with the proposed function for XB3.

**Figure 6:**
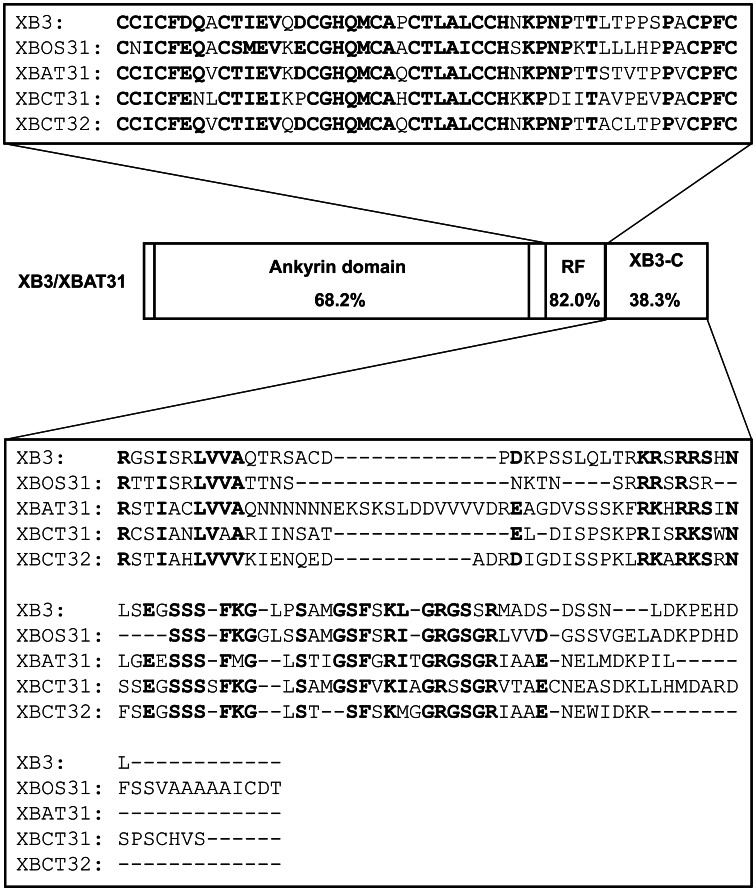
Schematic diagram showing structure and comparisons of the characterized protein family members in this study. Domain identities between the rice XB3 and the Arabidopsis XBAT31 are shown. Sequence alignments of the RING finger (RF) motif and the C-terminal tail (XB3-C) of the XB3 family members from rice (XB3 and XBOS31), Arabidopsis (XBAT31), and citrus (XBCT31 and XBCT32), are demonstrated. Identical amino acid residues are highlighted in bold.

We have previously shown that the N-terminal ankyrin repeats of XB3 are sufficient for interacting with the XA21 kinase region [14]. This study identifies the RF motif together with the XB3-C as the minimal region capable of triggering cell death. Therefore, the C-terminal half of XB3 likely acts as the signaling domain of this protein. Moreover, a single residue mutation (Cys323 to Ala) in the RF motif abolishes both cell death and E3 ubiquitin ligase activities [14]. Relative to other regions, the RF motif shares the highest identity (82.0%) between the rice XB3 and Arabidopsis XBAT31 (Figure 6). These collectively point to an essential role for the RF motif in the conserved cell death activity. Although the RF motif of XB3 possesses E3 ubiquitin ligase activity [14], agroinfiltration of a construct containing this domain failed to induce cell death. As evidenced by protein blot analysis, no product with the expected size of the RF motif accumulates in the infiltrated leaf discs. Instead, significant amounts of products with higher molecular weight were detected. In contrast, proteins with expected sizes were observed when expressing full-length XB3 or other truncation variants, despite the presence of higher molecular weight products in the XB3RFC and XB3Ank expressing leaves. These higher molecular weight products might represent a post-translational modification, such as ubiquitination, of the expressed truncation proteins by XB3 or other enzymes. Therefore, the failure of cell death activation by the *RF* construct could be attributed to the degradation of the RF protein.

XB3-mediated cell death may not only be a laboratory phenomenon, but could represent a conserved regulatory mechanism by which programmed cell death (PCD) is initiated. An XB3 family member, *MjXB3* from the four o’clock plant (*Mirabilis jalapa*), is induced 40,000-fold, in RT-PCR assays, during senescence of flower petals, a developmental process involving PCD [35]–[36]. Silencing a homolog of *MjXB3* in Petunia extends flower life by 20%. It has been suggested that *MjXB3* may be involved in regulating flower senescence, a developmental process involving PCD [35]–[37]. In line with this, analysis of publicly available microarray datasets reveals predominant expression of the Arabidopsis member *Xbat31* in senescing leaves, mature pollen and the second internode of stem [38]. Our data might therefore be interpreted as an assay that mimics leaf senescence. Because all five XB3 family members tested to date are capable of triggering cell death, it is highly likely that members of the XB3 family function as evolutionarily conserved cell death activators in the plant kingdom. The question of whether this cell death can lead to disease resistance remains to be addressed even though XB3 physically associates with XA21. It has been demonstrated that the Arabidopsis mitogen-activated protein kinase MPK6 plays a role in regulating both leaf senescence and defense [39]–[40]. This raises a possibility that members of the XB3 family might also be involved in modulating these two processes.

## Supporting Information

Table S1 Primers used in this study.
(DOCX)
