# Supplementary material for: Members of the XB3 Family from Diverse Plant Species Induce Programmed Cell Death in Nicotiana benthamiana
Source: PLoS One. 2013 May 22;8(5):e63868. doi: 10.1371/journal.pone.0063868 (PMC3661601; doi:10.1371/journal.pone.0063868)
Supplement: Table S1 — Primers used in this study. (DOCX) [file pone.0063868.s001.docx]

**Table 1 Primers used in this study**

**Name Sequence (5’-3’) Note**

Xb3nb-1 GGATCCACTAGTATGGGTCACGGTGTCAG For pC1300S-XB3 and

Xb3nb-2 GGATCCAGGATGATGCGGCGATTGTCCG pC1300S-XB3^C323A^

Xb3NEW-1 GTTCTAGAGGATCCATGGGTCACGGTGTCAGCTGCG For pCR8GW-XB3

Xb3NEW-3 TTTCTAGAAATCAACTAGTTAGATCGTGCTCAGGCTTGTCCA -3xFLAG

Xbos31nb-5 GTGTAGATCTAGAATGGGGCACGGCCTGAGCTGCA For pC1300S-

Xbos31nb-6 GTGTAGATCTTCAACTAGTTGTGTCACAAATAGCAGCAG XBOS31-3xFLAG

Xbos31nb-7 GTGCTCCCGGGGAATCCGTAGA For pC1300S- XBOS31-

3xFLAG sequencing

Xbat31nb-1 GTGTGGATCCCCTAGGATGGGGCAGAGTATGAGCTGTGGA For pC1300S-

Xbat31nb-2 GTGTGGATCCTCATCTAGACAATATTGGTTTGTCCATCAGCTCG XBAT31-3xFLAG

Xbat31nb-3 CGATCAGCACCCCAAGCAAGCA For pC1300S-XBAT31

-3xFLAG sequencing

Xbat32nb-1 GTGTGGATCCTCTAGAATGAGGTTTCTAAGCCTCGTCGGA For pC1300S-XBAT32

Xbat32nb-2 GTGTGGATCCTCAACTAGTGCAAGCACTTCCACCGGTTGTA -3xFLAG

Xbatnb-3 TGCGTGTTTCCACCACATGAAGCA For pC1300S-XBAT32

-3xFLAG sequencing

Xbct31nb-1 GTGGATCCATGGGTCAGAGAATGAGTTGTAGGGA For pC1300S-XBCT31

Xbct31nb-2 GTGGATCCTCAACTAACATGGCAAGAAGGAGAAT -3xFLAG

Xbct31nb-3 AGCAAGGCTGCACACGCTTGA For pC1300S-XBCT31

-3xFLAG sequencing

041054m-3 GGATCCATGGGTCAGGGACTGAGTTGTGGA For pC1300S-XBCT32

041054m-4 GGATCCTCAACTAGTACGCTTATCAATCCACTCATTTTCGG -3xFLAG

XB3New-1 GTTCTAGAGGATCCATGGGTCACGGTGTCAGCTGCG For pC1300S-XB3Ank

XB3New-5 GTTCTAGATCAACTAGTTGAGCATGCATCGTCATCGGCA -3xFLAG

XB3New-1 GTTCTAGAGGATCCATGGGTCACGGTGTCAGCTGCG For pC1300S- XB3ΔC

XB3New-7 GTTCTAGATCAACTAGTCGGCTTGTCAGGATCACAAGCAG - 3xFLAG

**Table 1 Primers used in this study**

**Name Sequence (5’-3’) Notes**

XB3New-6 GTTCTAGAGGATCCATGGCATGCTCAGAGGTGAGCGACA For pC1300S- XB3RFC

XB3CT-3 TTTCTAGAAATCAACTAGTTAGATCGTGCTCAGGCTTGTCCA -3xFLAG

XB3New-6 GTTCTAGAGGATCCATGGCATGCTCAGAGGTGAGCGACA For pC1300S- XB3RF

XB3New-7 GTTCTAGATCAACTAGTCGGCTTGTCAGGATCACAAGCAG -3xFLAG

XB3New-13 GTTCTAGAGGATCCATGGGCAGCATCTCACGGCTGGTGG For pC1300S- XB3C

XB3CT-3 TTTCTAGAAATCAACTAGTTAGATCGTGCTCAGGCTTGTCCA - 3xFLAG

1300SFLAG CCTGAGATCTCTAGAGTCGACCT For all FLAG-tagged

Constructs sequencing
